# Nitrogen and phosphorus fertilizer use efficiency improves alfalfa (*Medicago sativa* L.) production and performance in alkaline desert soil

**DOI:** 10.3389/fpls.2025.1526648

**Published:** 2025-02-18

**Authors:** Yanliang Sun, Jing Sun, Xuzhe Wang, Andrew D. Cartmill, Ignacio F. López, Chunhui Ma, Qianbing Zhang

**Affiliations:** ^1^ College of Animal Science and Technology, Shihezi University, Shihezi, Xinjiang, China; ^2^ School of Agriculture and Environment, Massey University, Palmerston North, New Zealand

**Keywords:** agronomic efficiency, best management practices (BMPs), environmental concerns, rational and economical fertilization, yield enhancement

## Abstract

The deficiency of nitrogen and phosphorus is a primary constraint on the normal growth of alfalfa (*Medicago Sativa* L.) in the alkaline desert soils of northern Xinjiang. Optimizing the combination of nitrogen and phosphorus fertilizers can maximally significantly enhance farmers’ economic returns while concurrently mitigate soil environmental pollution. For this purpose, a field experiment based on a randomized complete block design was conducted over two consecutive years (2019 and 2020) in Shihezi, Xinjiang province, China. The WL366HQ variety of alfalfa was evaluated with four levels each of urea and monoammonium phosphate. The effects of fertilizer treatments were assessed on alfalfa yield, growth traits, nutritional quality, fertilizer use efficiency, and economic benefit. Application of nitrogen (N), phosphorus (P), and their interaction significantly (*P*< 0.05) affected cumulative alfalfa dry matter (DM) yield. In general, compared to no-fertilization treatment, the application of N and P fertilizers resulted in increased plant height, stem thickness, crude protein, and ether extract of alfalfa, while neutral detergent fiber (NDF) and acid detergent fiber (ADF) exhibited a decreasing trend. Additionally, while N and P fertilizer application reduced corresponding fertilizer use efficiency, it increased non-corresponding fertilizer use efficiency. During the two-year experimental period, the treatment involving the application of urea at 286.3 kg·ha^-1^ combined with monoammonium phosphate at 192 kg·ha^-1^ achieved the highest evaluation scores for production performance, fertilizer use efficiency, and total net profit, resulting in a net profit increase of 44.18% compared to the no-fertilizer treatment. These findings lay the groundwork for nuanced fertilization strategies in future alfalfa cultivation.

## Graphical Abstract

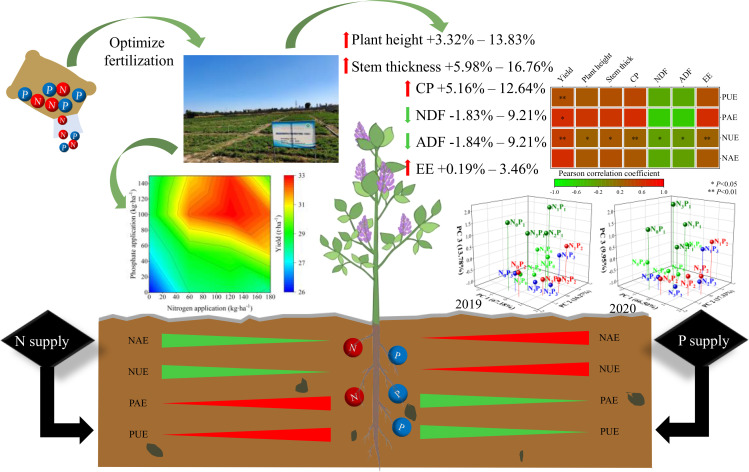

## 1 Introduction

Alfalfa (*Medicago sativa* L.) is a nutritionally rich and stress-resistant perennial legume widely used in various livestock production systems globally (Bhandari et al., 2020; Sun et al., 2022). Along with maize (Zea mays L.) silage, oats (Avena sativa L.), and ryegrass (Lolium perenne L.), alfalfa constitutes a primary forage crop for livestock in China. In the year 2021, China cultivated approximately 424,000 hectares of high-yielding alfalfa varieties, resulting in a total production of about 4.2 million tons, reflecting an 8.10% average yield increase per hectare compared to 2017. Despite these production gains, alfalfa imports remained substantial at around 1.78 million tons, marking a 30.93% year-over-year increase (Vurro et al., 2023; Xia et al., 2023). The rising demand for high-quality alfalfa requires improved nutrient and production efficiency, as well as the utilization of non-conventional resources, including degraded and sub-optimal lands. In China, alfalfa cultivation in arid and saline-alkaline regions like Xinjiang exemplifies such a practice. The alkaline desert soils in this region predominantly originate from loess-like diluvial-alluvial deposits with small gravel amounts. Soils are alkaline, prone to “alkaline whitening, hardening, and drying”, and have limited phosphorus (P) and nitrogen (N) availability, which negatively impacts crop production and performance, and thus the development and enhancement of the animal husbandry industry in this, and the surrounding regions (Liu et al., 2021).

Globally, crop growth is significantly affected by N and P limitations. Approximately 18% of arable land experiences N limitation, 43% suffers from P limitation, and the remaining 39% faces both N and P limitations (Putra et al., 2020). Nitrogen plays a crucial role in plant processes such as carbon and N metabolism, photosynthesis, and protein synthesis (Anas et al., 2020). Alfalfa forms symbiotic relationships with N-fixing bacteria called rhizobia, which inhabit nodules on its roots and convert atmospheric N into forms accessible to the plant. Despite this symbiosis, alfalfa may still suffer from N deficiency due to factors such as inadequate soil nutrient levels (Zielewicz et al., 2023), salt stress (Wan et al., 2023), and drought conditions (Liu et al., 2018). Some studies suggest that alfalfa can fix sufficient atmospheric N to meet its needs without additional N fertilizer (Zhou et al., 2019). However, other research indicates that N application can significantly enhance alfalfa yield, especially in nutrient-poor soils during initial planting, regrowth, and regreening phases (Elgharably and Benes, 2021). In China, alfalfa growers increasingly use N fertilizers as a practical strategy to boost yields, particularly in suboptimal conditions such as barren, arid areas including sandy and saline-alkaline soils (Liu et al., 2021; Wang et al., 2021a).

Along with N, P is crucial for plant growth and productivity, with crop yields highly dependent on soil P availability. P is involved in numerous cellular and growth processes, such as the maintenance and synthesis of membrane structures and biomolecules, enzyme activation and inactivation, and carbohydrate metabolism. These processes influence germination, photosynthesis, assimilate transport, shoot and root growth, flower and seed set, and overall yield (Chtouki et al., 2022; Malhotra et al., 2018). Research indicates that P availability can significantly impact alfalfa yields. Increased soil P enhances root development, accelerates plant growth, and enables early maturity and stress resistance, leading to higher yield and quality (Liu et al., 2021). Additionally, P availability affects N fixation in legumes. High N fertilizer rates tend to suppress root nodulation when P levels are low, whereas high P availability can promote nodulation even with substantial N application (Song et al., 2022).

Balancing crop yield, nutritional value, and environmental preservation is essential for maximizing economic value while minimizing ecological impact (Bastos et al., 2020). This study aims to investigate the effects of N and P fertilizer application on alfalfa production, fertilizer use efficiency, and economic benefit. The study hypothesizes that: (1) both N and P application promote alfalfa growth, which could increase the alfalfa growth rate and crude protein content; (2) N fertilizer application can improve P fertilizer use efficiency, and conversely, P fertilizer application can enhance N fertilizer use efficiency; and (3) increased N and P fertilizer use efficiency promotes increased alfalfa DM yield.

## 2 Materials and methods

### 2.1 Experimental site description

**Figure 1:**
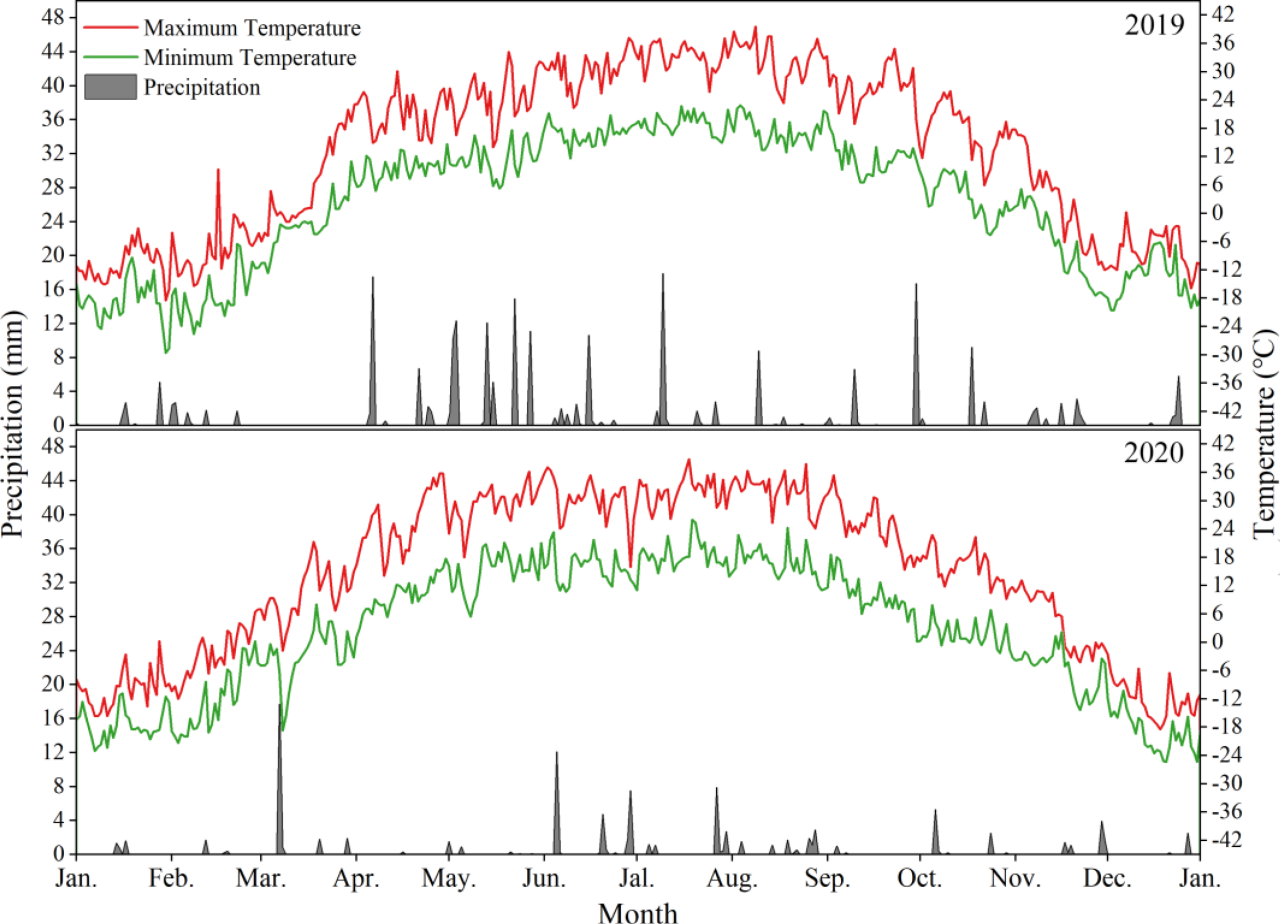
Daily precipitation, maximum and minimum temperatures during the experimental period in 2019 and 2020.

The study was conducted at the Modern Water-Saving Irrigation Corp Key Laboratory Test Base in Shihezi, Xinjiang province, China (altitude 420 m, coordinates 44°20’ N, 86°30’ E). It is situated on the northern slope of the Tianshan Mountains and on the southern edge of the Junggar Basin. The region features an arid temperate continental climate. In 2019 and 2020, annual rainfall was 231.3 mm and 103.8 mm, highest temperature was 39.6°C and 38.8°C, and lowest temperature was -29.6°C and -25.4°C, with an average annual temperature of 8.3°C and 8.7°C, respectively (**Figure 1**). The soil at the experimental site is characterized as alkaline desert soil, with detailed nutrient content provided in **Table 1**. Before this study, the fields were used for cultivating cotton (*Gossypium* spp.).

**Table 1:** Average values for soil characteristics of composite topsoil samples (0 - 20 cm) from the experimental fields.

Soil depth (cm)	Organic matter g·kg^−1^	Total nitrogen g·kg^−1^	Total phosphorus g·kg^−1^	Available potassium mg·kg^−1^	pH
0 - 20	21.56	1.18	0.53	119.80	7.95

### 2.2 Experimental design and management

The experiment was conducted as a two-factor randomized block design, with four N application levels (0, 60, 120, and 180 kg N·ha^-1^) and four P application levels (0, 50, 100, and 150 kg P_2_O_5_·ha^-1^). The experiment was repeated three times, resulting in a total of 48 plots. Urea fertilizer (with N content ≥ 46%) and monoammonium phosphate (with P_2_O_5_ content ≥ 52% and N content ≥ 12.2%) were used as N and P fertilizers, respectively. Due to the presence of a small amount of N in monoammonium phosphate, additional N was supplemented to maintain consistent N levels among different P application treatments under the same N application conditions. Specifically, the urea added for treatments P_0_, P_1_, P_2_, and P_3_ was 76.5, 51.0, 25.5, and 0 kg·ha^-1^, respectively (**Table 2**). Irrigation during each annual growing season totaled approximately 6750 m^3^·ha^-1^. Both N and P fertilizers were applied using drip irrigation over a period of 3-5 days during the alfalfa re-greening stage and after each of the first three cuts. Harvest dates were July 14th, August 19th, and September 29th, 2019, and May 25th, July 4th, August 14th, and October 4th, 2020.

**Table 2:** Nutrient content of the different fertilization treatments.

Treatment	Monoammonium phosphate (P_2_O_5_≥52%, N≥12.2%) kg·ha^-1^	Urea (N≥46%) kg·ha^-1^	P_2_O_5_ kg·ha^-1^	N kg·ha^-1^
N_0_P_0_	0	76.3	0	35.1
N_0_P_1_	96	50.8	50	35.1
N_0_P_2_	192	25.4	100	35.1
N_0_P_3_	288	0	150	35.1
N_1_P_0_	0	206.7	0	95.1
N_1_P_1_	96	181.2	50	95.1
N_1_P_2_	192	155.8	100	95.1
N_1_P_3_	288	130.4	150	95.1
N_2_P_0_	0	337.2	0	155.1
N_2_P_1_	96	311.7	50	155.1
N_2_P_2_	192	286.3	100	155.1
N_2_P_3_	288	260.9	150	155.1
N_3_P_0_	0	467.6	0	215.1
N_3_P_1_	96	442.1	50	215.1
N_3_P_2_	192	416.7	100	215.1
N_3_P_3_	288	391.3	150	215.1

The alfalfa variety used in the experiment was WL366, with a fall dormancy rating of 5. In April 2019, the alfalfa was sown at a rate of 18.0 kg·ha^-1^, using a manual row seeding method. Row spacing was 20 cm, and the sowing depth was 2 cm. Drip tapes was spaced at 60 cm intervals, and shallowly buried at a depth of 10 cm below the soil surface. Plots were 24 m^2^ (4 m × 6 m) in size, with a 1 m border between each plots to prevent water and nutrient infiltration. Besides fertilization, practices for irrigation, weed control, and pest management were consistent with those used in local high-yielding fields.

### 2.3 Plant sampling, measurement, and calculations

During the early flowering stage (5%-10% flowering), 1 m2 of alfalfa samples were randomly selected and harvested (stubble height 5 cm), from each experimental plot and fresh mass (FM) was determination. An additional ~400 g of samples were collected and dried at 105°C for 30 min and then at 65°C until constant mass was achieved. Moisture content was calculated and subsequently convert it to DM yield. Plant height and stem diameter (5 cm above soil surface) were also determined from 10 randomly selected plants. Total N was determined by the Kjeldahl method after H2SO4-H2O2 digestion pre-treatment, and crude protein (CP) was calculated by multiplying total nitrogen by 6.25. Neutral detergent fiber (NDF) and acid detergent fiber (ADF) were determined using the Van Soest method (Van Soest et al., 1991), and ether extract (EE) was determined using the ether extraction method (AOAC, 2005). Total P was determined by the molybdenum blue method after ashing pre-treatment (Fan et al., 2011). Nutrient agronomic use efficiency (NUAE) and nutrient uptake efficiency (NUUE) (Lin et al., 2022) were calculated by the following formula:


(1) $$\begin{array}{l} Nutrient\;agronomic\;use\;efficiency\\ =\frac{\left(yield\;with\;feretilizer-yield\;without\;fertilizer\right)}{fertilizer\;application} \end{array}$$

(2) $$Nutrient\;uptake\;efficiency=\frac{plant\;nutrient\;uptake}{fertilizer\;application}$$

### 2.4 Statistical analyses

Statistical analyses including analysis of variance (ANOVA), Z-Score standardization, and principal component analysis (PCA) were conducted using SPSS 27 (SPSS Inc., Chicago, IL, USA). A two-way ANOVA was conducted to investigate the impact of N and P on alfalfa growth and performance. Subsequently, multiple comparisons were carried out using Duncan’s technique. Prior to ANOVA, data was evaluate for normality using the Shapiro-Wilk test, and homogeneity using Bartlett’s test. Pearson correlation coefficients were calculated to analyze the relationships among different variables in alfalfa, including yield, growth traits, and nutritional quality. The correlation between nutrient agronomic use efficiency and nutrient uptake efficiency with yield was evaluated using the model y=ax+b . Graphs were generated using Origin Pro 2022 (Origin Lab, Northampton, MA, USA).

## 3 Result

### 3.1 Cumulative yield

**Figure 2:**
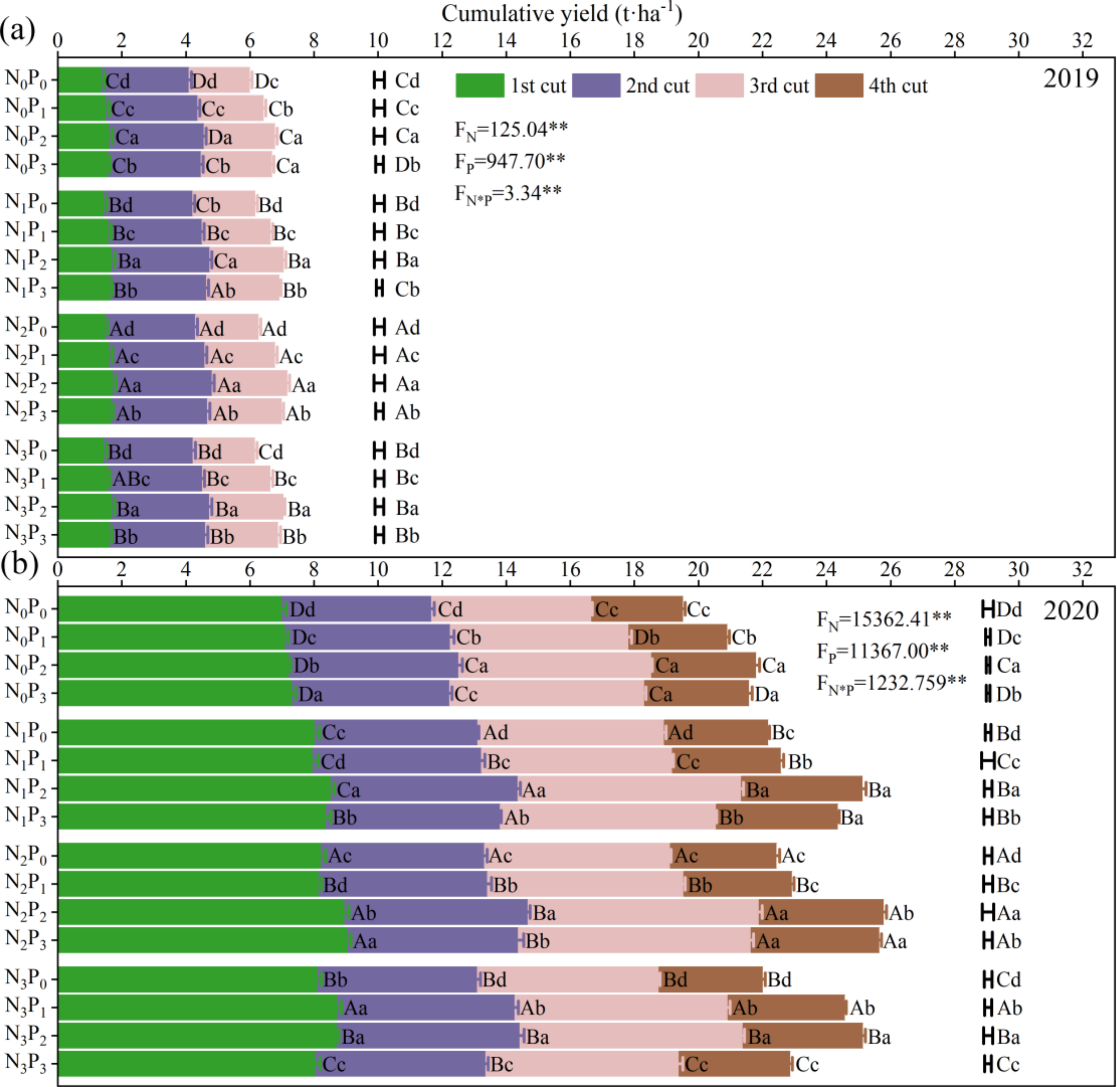
Effect of nitrogen (N) and phosphorus (P) treatments on the cumulative yield of alfalfa at different cuts (harvests) in 2019 **(A)** and 2020 **(B)**. Data are presented as the mean ± SD (n = 3). The previous error bars and letters are the standard deviation and multiple comparison results of each cut of alfalfa yield, and the last column of error bars and letters are the standard deviation and multiple comparison results of the annual cumulative yield. Different capital letters indicate significant (*P*< 0.05) differences between different N fertilizer levels under the same P application condition (Different small letters indicate significant (*P*< 0.05) differences between different P fertilizer treatments under the same N application condition. F_N_, F_P_ and F_N_ × F_P_ represent the F value under the N application levels, P application levels and the interaction of N and P application levels, respectively. ** indicates extremely significant (*P*< 0.01) difference.

A significant difference in cumulative DM yield was observed between 2019 and 2020, with yields notably lower in 2019 (the first year of planting) compared to 2020. The N application level, P application level, and their interaction level had a significant (*P<* 0.01) effects on the cumulative yield of alfalfa in both years (**Figure 2**). The cumulative yield ranged from 6.02 to 7.20 t·ha^-1^ in 2019 and from 19.54 to 25.80 t·ha^-1^ in 2020. Under the same N treatment, an increase in P application showed a trend of initially increasing the cumulative yield and then decreasing overtime. Except for the P_1_ treatment in 2020, other P treatments exhibited a similar trend of initially increasing and then decreasing the cumulative yield of alfalfa with increased N application.

### 3.2 Growth trait

**Figure 3:**
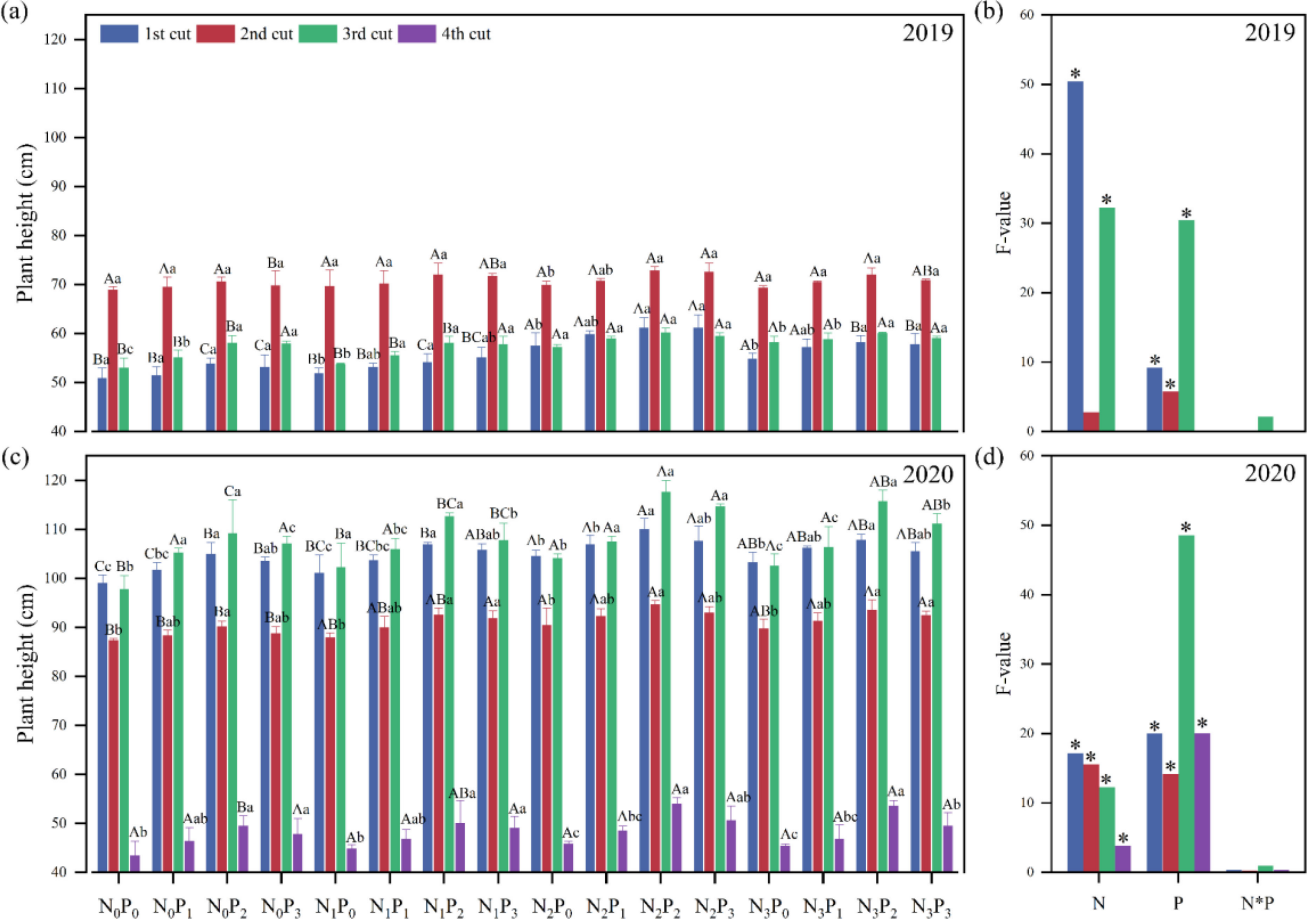
Effect of nitrogen (N) and phosphorus (P) treatments on the plant height of alfalfa at different cuts in 2019 **(A, B)** and 2020 **(C, D)**. Data are presented as the mean ± SD (n = 3). Different capital letters indicate significant (*P*< 0.05) differences between different N fertilizer levels under the same P application condition. Different small letters indicate significant (*P*< 0.05) differences between different P fertilizer treatments under the same N application condition. * indicates significant (*P*< 0.05) difference.

Plant height is one of the indicators that characterize crop morphology and the main expression of above-ground growth response of crop to water and nutrients. Except for the N level applied to the second cut in 2019, there were significant (*P*< 0.05) differences in alfalfa plant height between N and P levels applied (**Figures 3B, D**). In 2019, plant height was significantly (*P*< 0.05) greater in P_2_ treatment than in P_0_ treatment under the same nitrogen application conditions, except for the first cut at N_0_ level, the second cut at N_0_, N_1_ and N_3_ levels, and the third cut at N_2_ level. Plant height was significantly (*P*< 0.05) greater in the N_2_ treatment than in the N_0_ treatment under the same P application conditions, except for the P_3_ level in the second and third cuts (**Figure 3A**). In 2020, under the same P conditions, the plant height of the N_2_ treatment was significantly (*P*< 0.05) higher than the N_0_ treatment, except for the N_2_ level in the fourth cut. Plant height of the P_2_ treatment was significantly (*P*< 0.05) greater than the P_0_ treatment under the same N application conditions, except for the P_1_ level in the third cut and at the P_0_, P_1_, and P_3_ levels in the fourth cut (**Figure 3C**).

**Figure 4:**
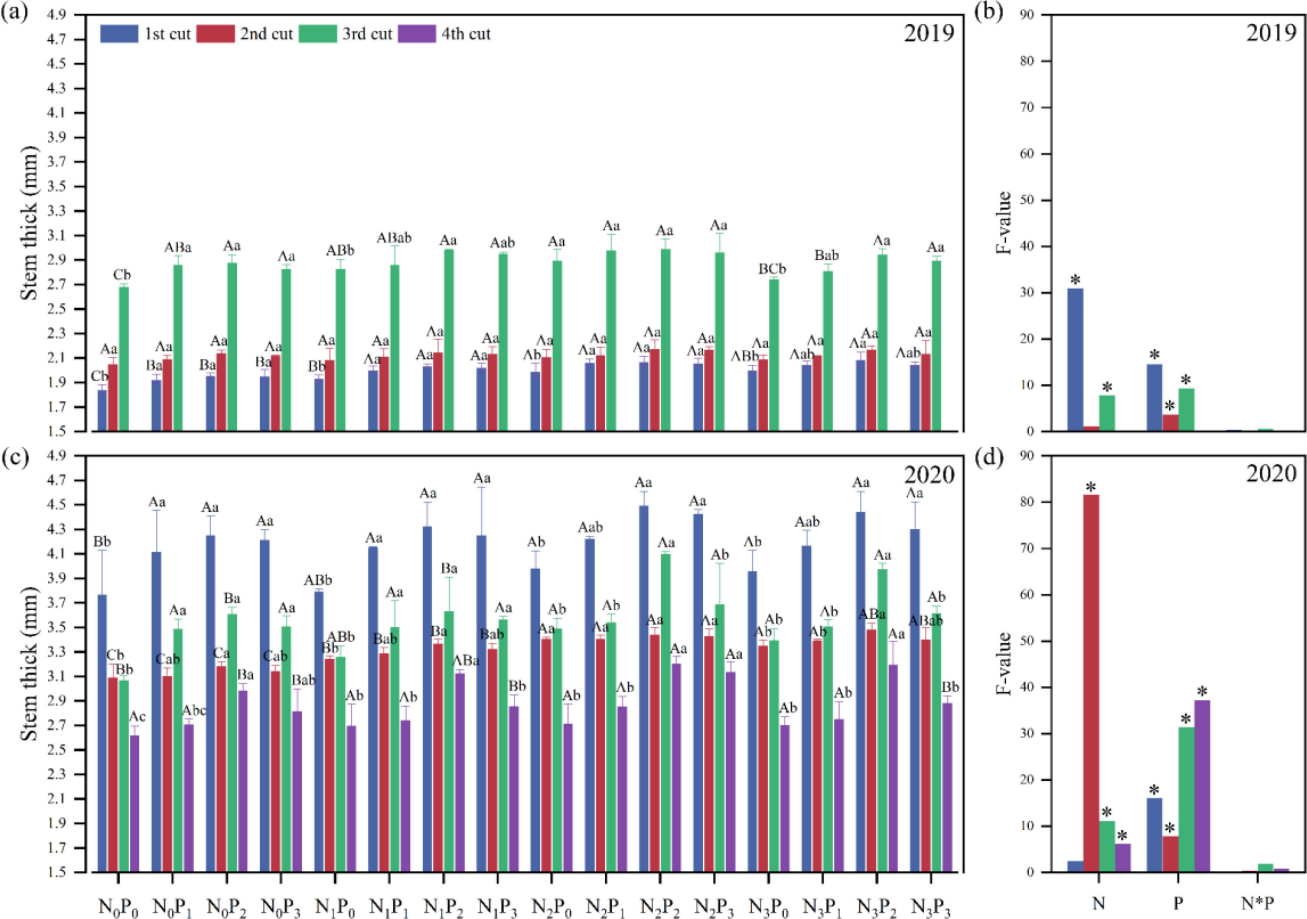
Effect of nitrogen (N) and phosphorus (P) treatments on stem thick of alfalfa at different cuts in 2019 **(A, B)** and 2020 **(C, D)**. Different capital letters indicate significant (*P*< 0.05) differences between different N fertilizer levels under the same P application. Different small letters indicate significant (*P*< 0.05) differences between different P fertilizer treatments under the same N application. * indicates significant (*P*< 0.05) difference.

Stems are the main support organ of plants, and stem thickness is closely related to the lodging resistance and crop yield. Except for the second cut in 2019 and the first cut in 2020, there were significant (*P*< 0.05) differences in stem thickness between different levels of N and P application (**Figures 4B, D**). In 2019, under the same N conditions, the stem thickness under the P_2_ treatment was significantly (*P*< 0.05) greater than the P_0_ treatment, except for the second and third cuts at the N_2_ level. Under the same phosphorus conditions, the stem thickness under the N_2_ treatment was significantly (*P*< 0.05) greater than that under the N_0_ treatment, except for the second and third cuts at the P_2_ and P_3_ levels (**Figure 4A**). In 2020, under the same N conditions, the stem thickness under the P_2_ treatment was significantly (*P*< 0.05) greater than the P_0_ treatment, except for the second cut at the N_2_ level. Under the same N conditions, the stem thickness under the N_2_ treatment was significantly (*P*< 0.05) greater than the N_0_ treatment, except for the first, second, and third cuts at P_1_, P_2_, and P_3_ levels, and the fourth cut at the P_0_ and P_1_ levels (**Figure 4C**).

### 3.3 Nutritional quality

**Figure 5:**
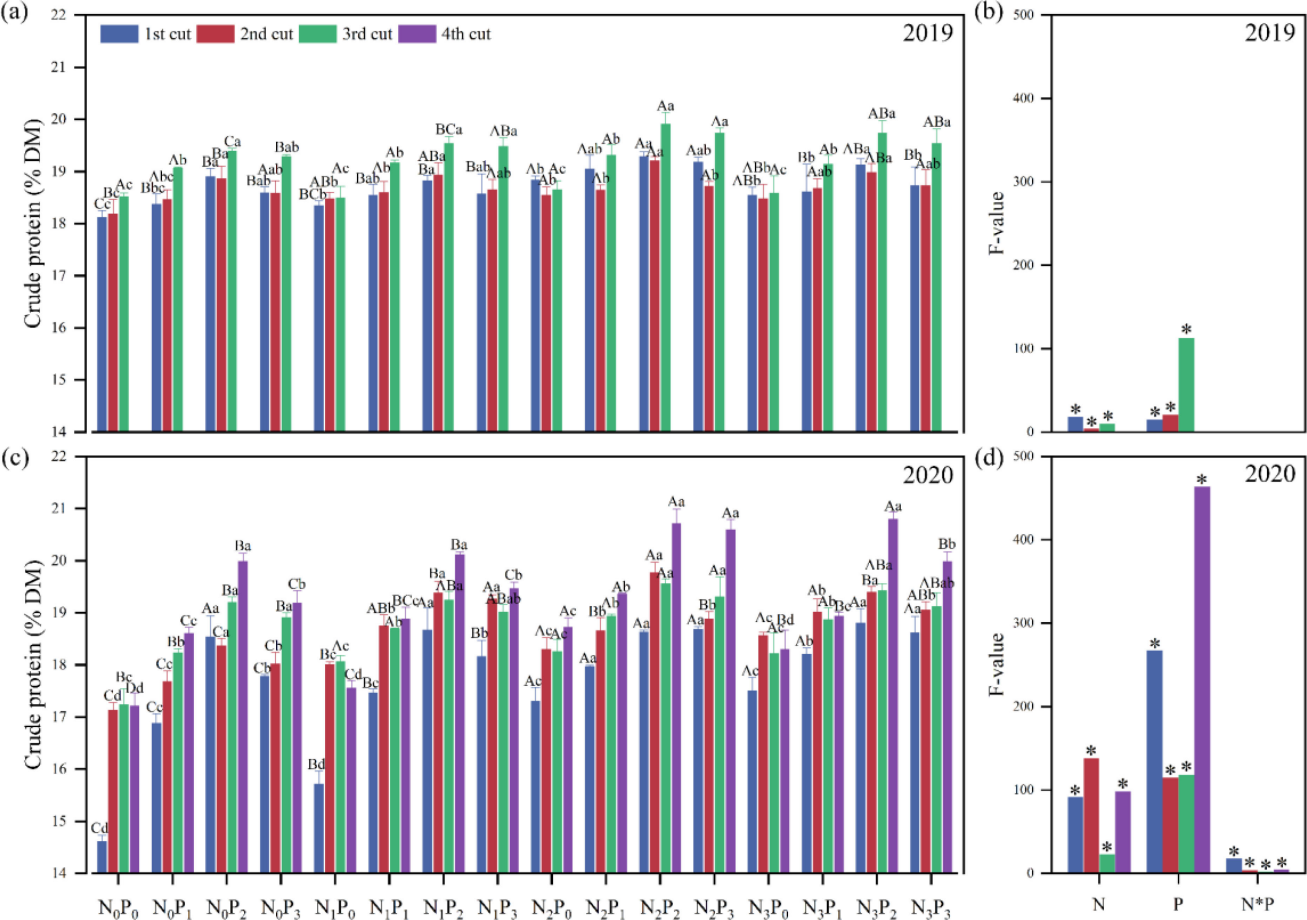
Effect of nitrogen (N) and phosphorus (P) treatments on the crude protein (CP) of alfalfa at different cuts in 2019 **(A, B)** and 2020 **(C, D)**. Different capital letters indicate significant (*P*< 0.05) differences between different N fertilizer levels under the same P application condition. Different small letters indicate significant (*P*< 0.05) differences between different P fertilizer treatments under the same N application condition. * indicates significant (*P*< 0.05) difference.

Crude protein (CP), NDF, ADF, and EE directly reflect the nutritional quality of alfalfa. There were significant (*P*< 0.05) differences in alfalfa CP by N application level, P application level, and N-P interaction level, except for the N-Pinteraction level in 2019 (**Figures 5B, D**). There were significant differences in CP of alfalfa among different levels of N application, P application, and N-P interaction (*P*< 0.05), except for the N-P interaction level in 2019 (**Figures 5B, D**). In both 2019 and 2020, under the same N conditions, the CP under the P_2_ treatment was significantly (*P*< 0.05) greater than the P_0_ treatment (**Figures 5A, C**). In 2019, under the same P conditions, the CP under the N_2_ treatment was significantly (*P*< 0.05) greater than the N_0_ treatment, except for the first and third cuts at the P_1_ and P_3_ levels, and the second and third cuts at the P_0_ and P_1_ levels (**Figure 5A**). In 2020, under the same P conditions, the CP under the N_2_ treatment was significantly (*P*< 0.05) greater than the N_0_ treatment, except for the second cut at the P_2_ level (**Figure 5C**).

**Figure 6:**
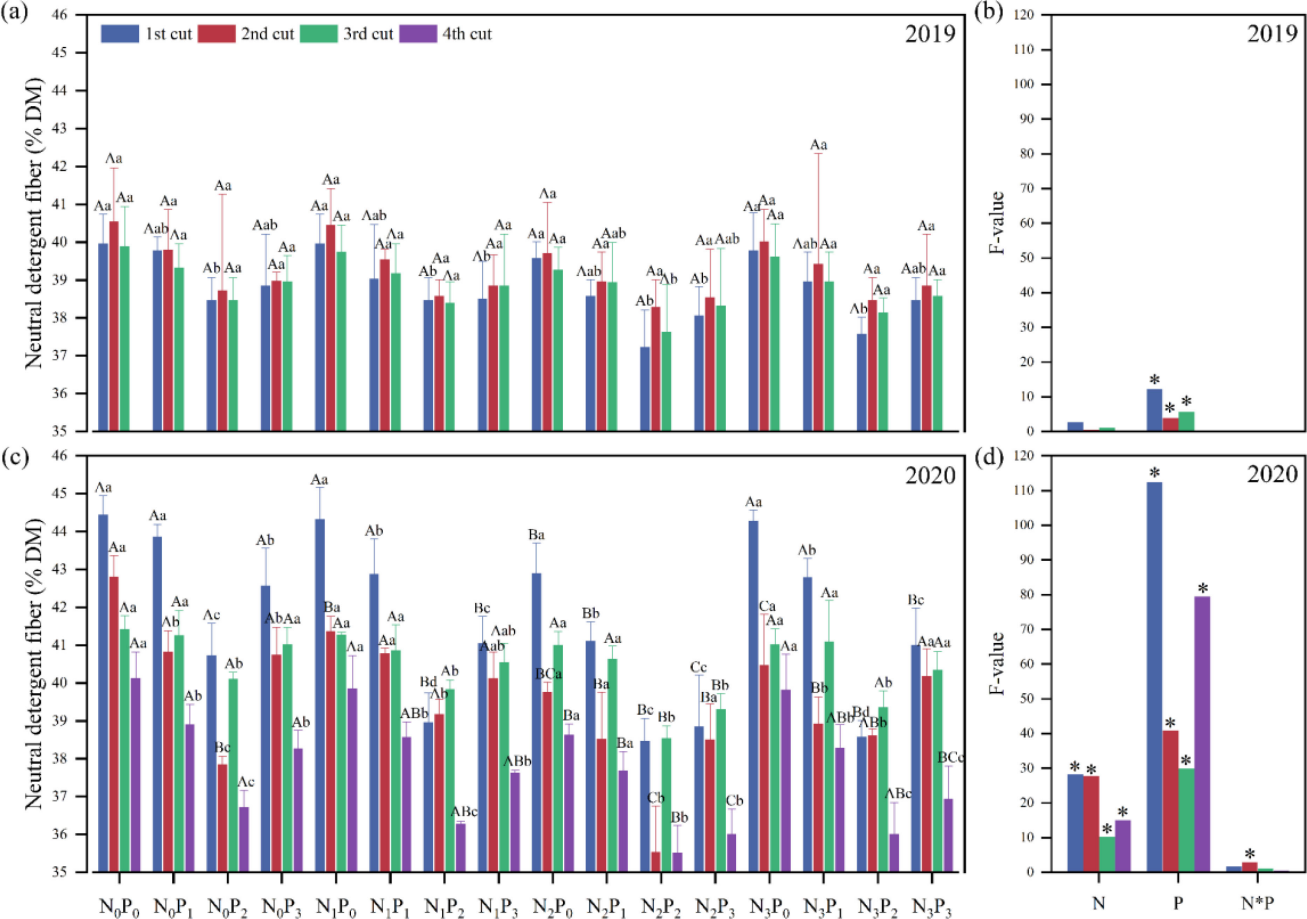
Effect of nitrogen (N) and phosphorus (P) treatments on the neutral detergent fiber (NDF) of alfalfa at different cuts in 2019 **(A, B)** and 2020 **(C, D)**. Different capital letters indicate significant (*P*< 0.05) differences between different N fertilizer levels under the same P application condition. Different small letters indicate significant (*P*< 0.05) differences between different P fertilizer treatments under the same N application condition. * indicates significant (*P*< 0.05) difference.

In 2019, the P application level and in 2020, the N application level, P application level, and N-P interaction level in the second cut showed significant (*P*< 0.05) differences in neutral detergent fiber (NDF) of alfalfa (**Figures 6B, D**). In 2019, under the same phosphorus level, there was no significant difference in NDF among different nitrogen treatments (*P* > 0.05). For the first and third cuts under N_2_ conditions, under the same nitrogen level, the NDF under the P_0_ treatment was significantly (*P*< 0.05) greater than the P_2_ treatment (**Figure 6A**). In 2020, except for the third cut at the P_0_ and P_1_ levels, under the same P conditions, the NDF under the N_0_ treatment was significantly (*P*< 0.05) higher than the N_2_ treatment (*P*< 0.05). Under the same N conditions, the NDF under the P_0_ treatment was significantly (*P*< 0.05) greater than the P_2_ treatment (**Figure 6C**).

**Figure 7:**
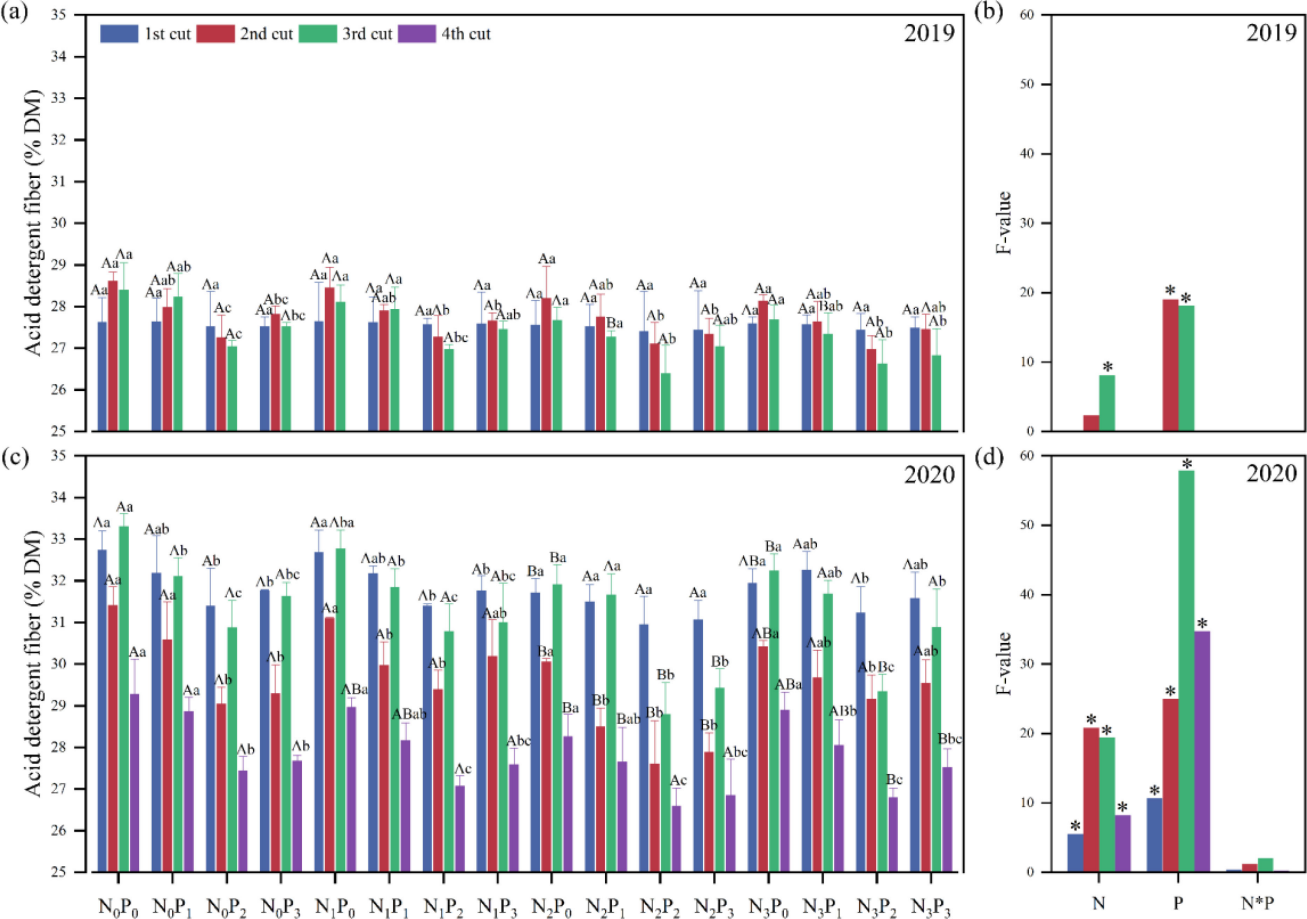
Effect of nitrogen (N) and phosphorus (P) treatments on the acid detergent fiber (ADF) of alfalfa at different cuts in 2019 **(A, B)** and 2020 **(C, D)**. Different capital letters indicate significant (*P*< 0.05) differences between different N fertilizer levels under the same P application condition. Different small letters indicate significant (*P*< 0.05) differences between different P fertilizer treatments under the same N application condition. * indicates significant (*P*< 0.05) difference.

In 2019, the N application level in the second cut, P application level in the second and third cuts, and in 2020, the N application level, P application level showed significant (*P*< 0.05) differences in acid detergent fiber (ADF) of alfalfa (**Figures 7B, D**). In the third cut of 2019 at the P_3_ level, under the same P conditions, the ADF under the N_0_ treatment was significantly (*P*< 0.05) greater than the N_2_ treatment. For the second and third cuts, under the same N conditions, the ADF under the P_0_ treatment was significantly (*P*< 0.05) greater than the P_2_ treatment (**Figure 7A**). In 2020, except for the first cut at the P_1_, P_2_, and P_3_ levels, the third cut at the P_1_ level, and the fourth cut at the P_2_ and P_3_ levels, under the same P conditions, the ADF under the N_0_ treatment was significantly (*P*< 0.05) greater than the N_2_ treatment. Except for the N_2_ level in the first cut of 2020, under the same N conditions, the ADF under the P_0_ treatment was significantly (*P*< 0.05) greater than that under the P_2_ treatment (**Figure 7C**).

**Figure 8:**
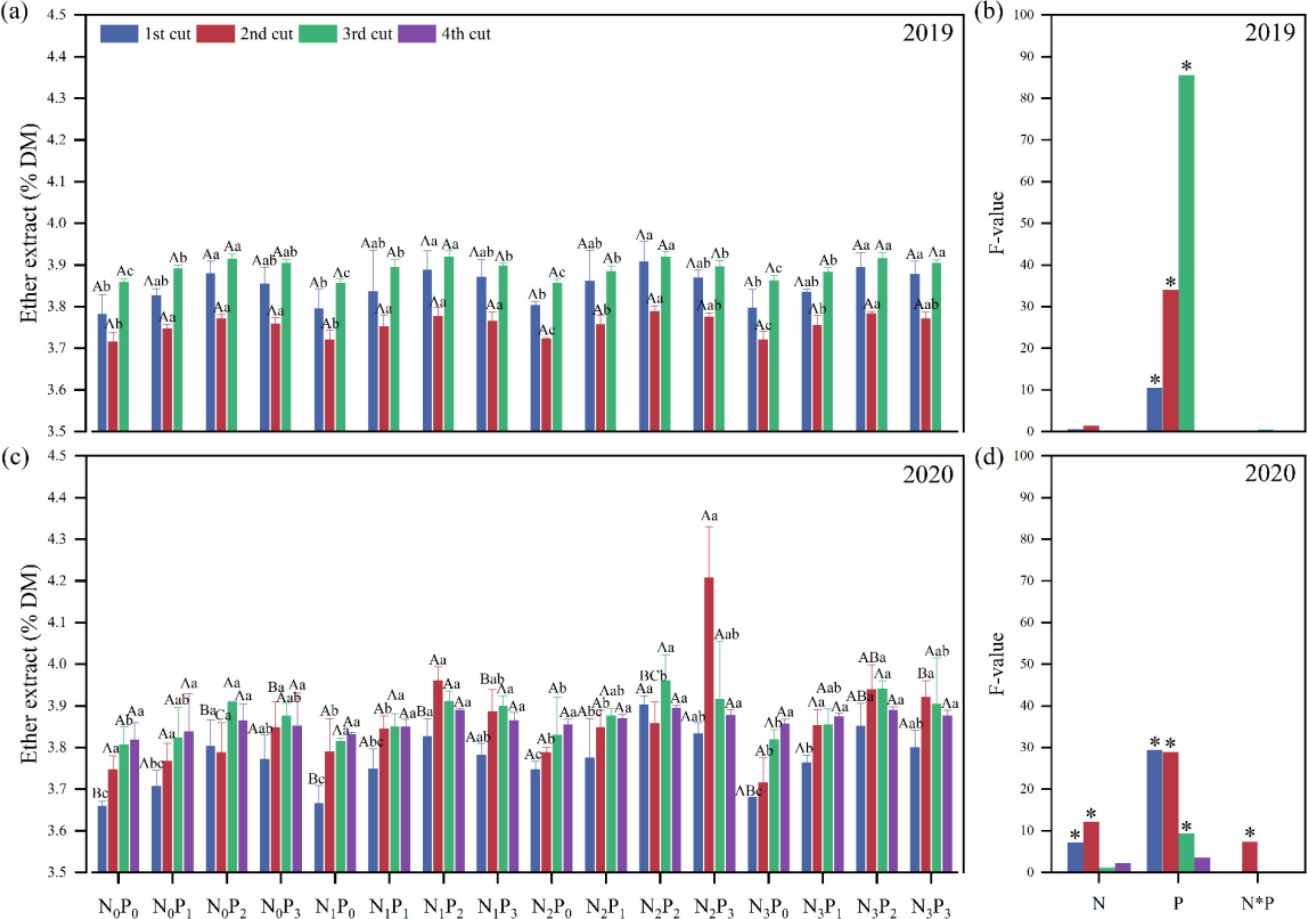
Effect of nitrogen (N) and phosphorus (P) treatments on the ether extract (EE) of alfalfa at different cuts in 2019 **(A, B)** and 2020 **(C, D)**. Different capital letters indicate significant (*P*< 0.05) differences between different N fertilizer levels under the same P application condition. Different small letters indicate significant (*P*< 0.05) differences between different P fertilizer treatments under the same N application condition. * indicates significant (*P*< 0.05) difference.

In 2019, the P application level, the N application level in the first two cuts of 2020, the P application level in the first three cuts, and the N-P interaction level in the second cut showed significant (*P*< 0.05) differences in ether extract (EE) of alfalfa (**Figures 8B, D**). Under the same P level in 2019, there was no significant difference in EE among different N treatments. However, under the same N conditions, the EE under the P_0_ treatment was significantly (*P*< 0.05) greater than that under the P_2_ treatment (**Figure 8A**). In 2020, the first cut at the P_0_ and P_2_ levels, the second cut at the P_2_ and P_3_ levels, under the same P conditions, the EE under the N_0_ treatment was significantly (*P*< 0.05) greater than the N_2_ treatment. Except for the N_0_ level in the second cut and various levels in the fourth cut, under the same N conditions, the EE under the P_0_ treatment was significantly (*P*< 0.05) greater than the P_2_ treatment (**Figure 8C**).

### 3.4 Correlation matrix between dry matter yield, growth traits and nutritional quality of alfalfa

**Figure 9:**
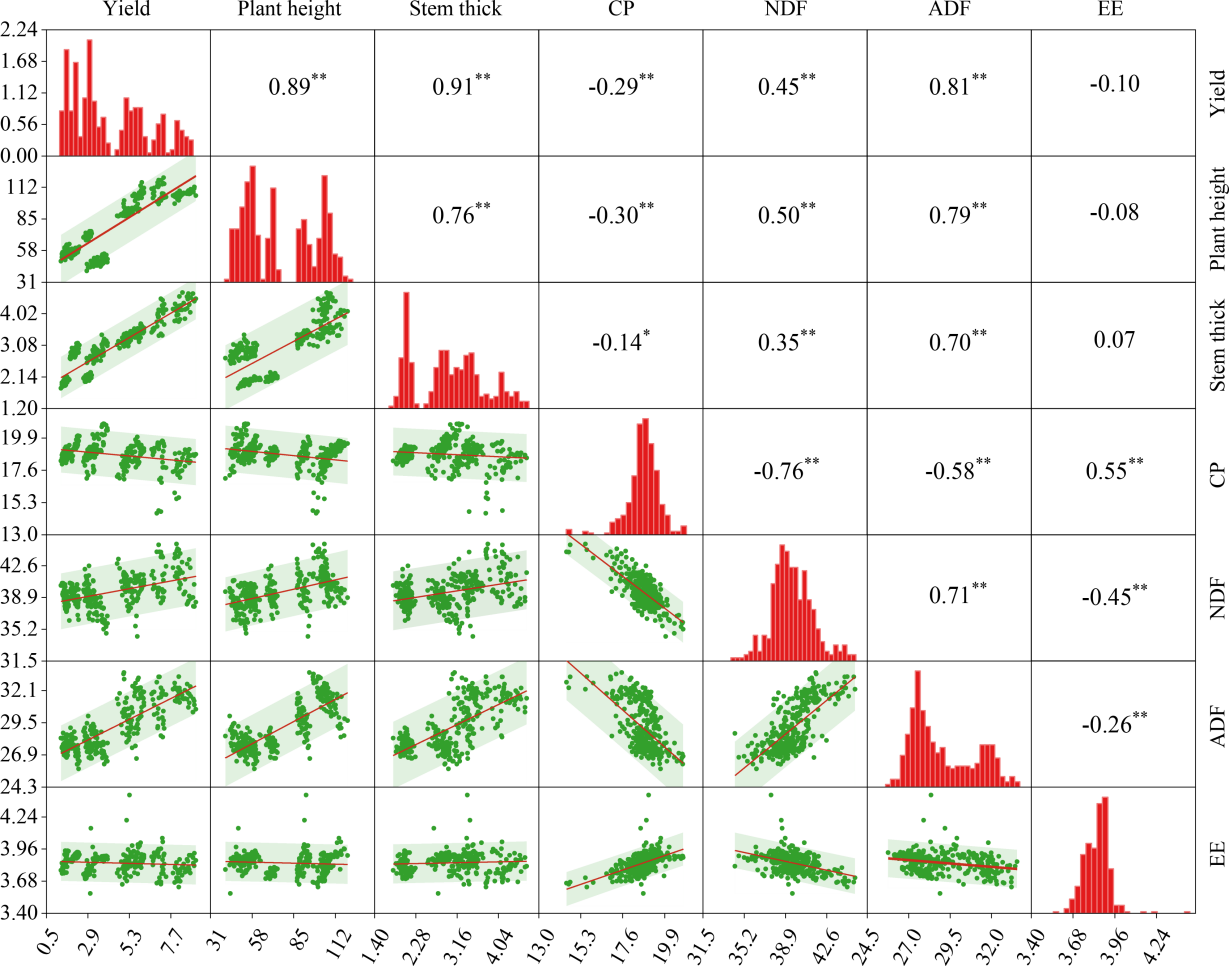
Correlation matrix between dry matter yield (DM), growth traits, and nutritional quality of alfalfa. The bottom left lines are least-squares fits and the shaded areas are the 95% CI regions, and the top right is the Pearson correlation coefficient. * indicates significant difference at *P*< 0.05 and ** indicates extremely significant difference at *P*< 0.01.

Further correlation analysis was conducted on DM yield, growth traits (plant height, stem thickness), and nutritional quality (CP, NDF, ADF, and EE) of alfalfa under different fertilizer treatments (**Figure 9**). Dry matter yield of alfalfa showed significant (*P<* 0.01) positive correlations with plant height, stem thickness, NDF, and ADF, while it exhibited a significant (*P<* 0.01) negative correlation with CP. CP showed a significant (*P<* 0.01) positive correlation with EE, but it had a significant negative correlations with plant height, NDF, ADF, and a significant (*P<* 0.05) negative correlation with stem thickness. Plant height showed significant (*P<* 0.01) positive correlations with stem thickness, NDF, and ADF (*P<* 0.01). Stem thickness had a significant (*P<* 0.01) positive correlations with NDF and ADF (*P<* 0.01). NDF showed a significant (*P<* 0.01) negative correlation with ADF, while EE had a significant (*P<* 0.01) negative correlations with both NDF and ADF.

### 3.5 Fertilizer agronomic use efficiency and fertilizer uptake efficiency of alfalfa under different fertilization treatments

**Figure 10:**
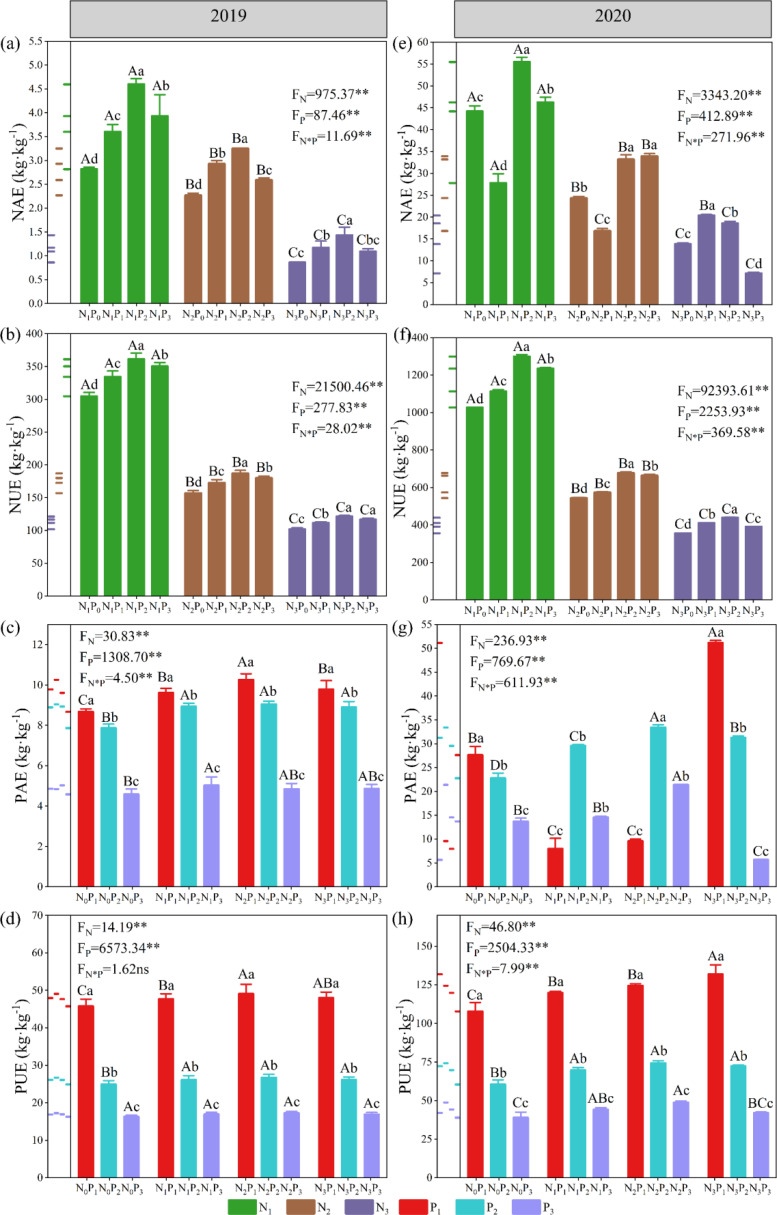
Fertilizer agronomic use efficiency and fertilizer uptake efficiency of alfalfa under different fertilization treatments in 2019 **(A–D)** and 2020 **(E–H)**. NAE: nitrogen (N) agronomic use efficiency; NUE: N uptake efficiency; PAE: phosphorus (P) agronomic use efficiency; and PUE: P uptake efficiency. Data are presented as the mean ± SD (n = 3). Different capital letters indicate significant (*P*< 0.05) differences between different N fertilizer levels under the same P application condition. Different small letters indicate significant (*P*< 0.05) differences between different P fertilizer treatments under the same N application condition (*P*< 0.05). F_N_, F_P_ and F_N_ × F_P_ represent the F value under the N application levels, P application levels and the interaction of N and P application levels, respectively. ns indicates no significant (*P* > 0.05) difference and ** indicates significant (*P* > 0.01) difference.

We calculated the fertilizer use efficiency during the experimental period and found that, except for the NAE at the N_1_ level in 2020, under the same P conditions, both NAE and NUE showed a significant (*P*< 0.05) decrease with increasing N application. Except for the NAE under the N_1_ and N_2_ conditions in 2020, under the same N conditions, both NAE and NUE showed an initial increase followed by a decrease with increasing P application (**Figures 10A, B, E, F**). Except for the PAE under the N_1_ and N_2_ conditions in 2020, under the same N conditions, both PAE and PUE showed a significant (*P*< 0.05) decrease with increasing P application. Except for the PAE and PUE under the P_1_ condition in 2020, under the same P conditions, both PAE and PUE showed an increase followed by a decrease with increasing N application (**Figures 10C, D, G, H**).

### 3.6 Regression relationship between fertilizer agronomic use efficiency, fertilizer uptake efficiency and cumulative yield

**Figure 11:**
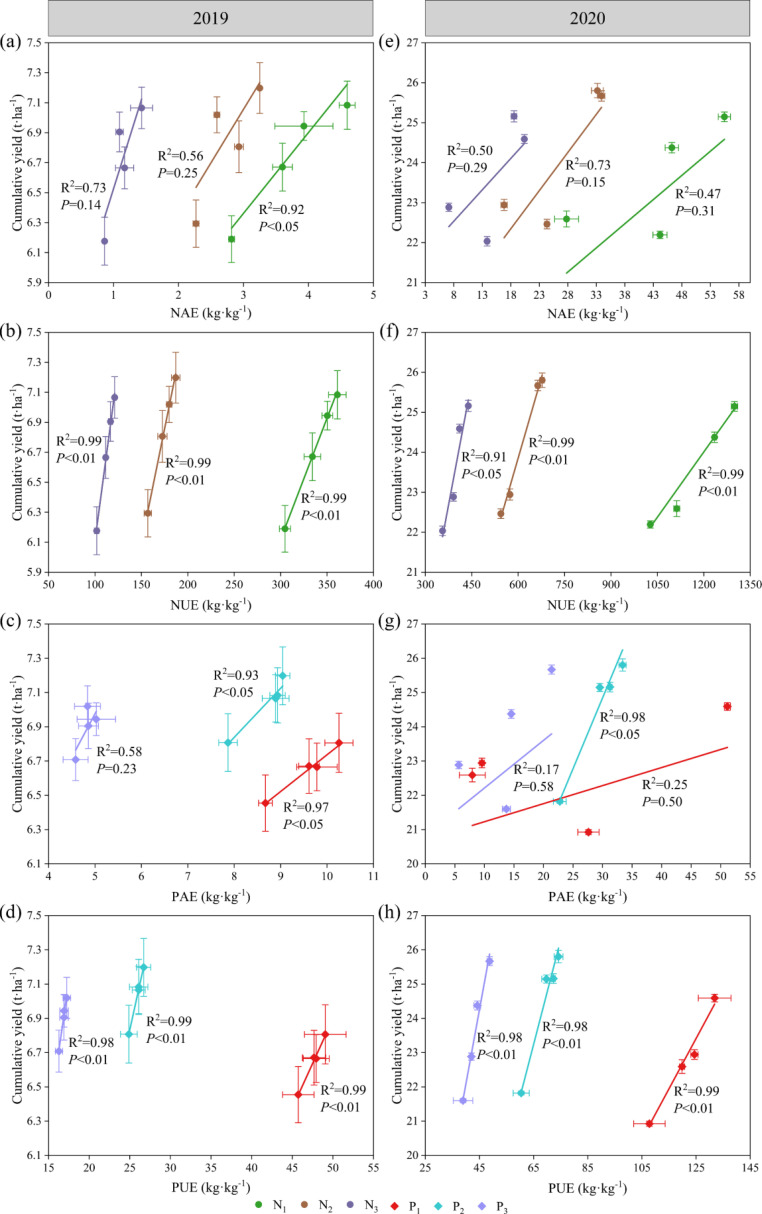
Regression relationship between cumulative yield and nitrogen (N) agronomic use efficiency **(A, E)**, N uptake efficiency **(B, F)**, phosphorus (P) agronomic use efficiency **(C, G)**, and P uptake efficiency **(D, H)** in 2019 and 2020. NAE, N agronomic use efficiency; NUE, N uptake efficiency; PAE, P agronomic use efficiency; PUE, P uptake efficiency.

A linear regression analysis was performed to examine the relationship between cumulative yield of alfalfa and different fertilizer use efficiency indicators. The results yielded first-order response models between cumulative yield and NAE, NUE, PAE, and PUE. It was found that the fitting coefficients between cumulative yield and NUE, PUE were higher than those between cumulative yield and NAE, PAE. The models between cumulative yield and NUE exhibited statistical significance, with R^2^ values above 0.91. Similarly, the models between cumulative yield and PUE also showed statistical significance, with R^2^ values above 0.98. This indicates that NUE and PUE can explain more than 90% of the yield data (**Figures 11B, D, F, H**). The regression coefficients between cumulative yield and NAE were all above 0.47, but only the cumulative yield under the N_1_ condition in 2019 showed statistical significance with NAE. The regression coefficients between cumulative yield and PAE were all above 0.17, with statistical significance observed for cumulative yield under the P_1_ and P_2_ conditions in 2019 and the P_2_ condition in 2020 (**Figures 11A, C, E, G**).

### 3.7 Principal component analysis

For further comprehensive evaluation of production performance, nutrient quality and fertilizer use efficiency of different fertilization treatments. The mathematical model for the comprehensive evaluation of principal component analysis in 2019 was as follows:


(3) $$\begin{array}{c} f_{1}=0.134\times Z_{1}+0.115\times Z_{2}+0.129\times Z_{3}+0.133\times Z_{4}-0.132\\ \times Z_{5}-0.128\times Z_{6}+0.128\times Z_{7}+0.064\times Z_{8}+0.042\times Z_{9}\\ +0.102\times Z_{10}+0.069\times Z_{11} \end{array}$$

(4) $$\begin{array}{c} f_{2}=-0.010\times Z_{1}-0.006\times Z_{2}+0.107\times Z_{3}-0.049\times Z_{4}+0.067\\ \times Z_{5}+0.094\times Z_{6}-0.102\times Z_{7}+0.469\times Z_{8}+0.500\times Z_{9}\\ -0.131\times Z_{10}-0.132\times Z_{11} \end{array}$$

(5) $$\begin{array}{c} f_{3}=-0.019\times Z_{1}-0.249\times Z_{2}-0.069\times Z_{3}-0.119\times Z_{4}+0.146\\ \times Z_{5}+0.189\times Z_{6}-0.015\times Z_{7}+0.102\times Z_{8}+0.128\times Z_{9}\\ +0.463\times Z_{10}+0.612\times Z_{11} \end{array}$$

(6) $$F_{2019}=A_{1}\times f_{1}+A_{2}\times f_{2}+A_{3}\times f_{3}$$

In the mathematical model, *f*_1_, *f*_2_, and *f*_3_ represent the scores of principal components 1, 2, and 3, respectively, in 2019. *Z*_1_, *Z*_2_, *Z*_3_, *Z*_4_, *Z*_5_, *Z*_6_, *Z*_7_, *Z*_8_, *Z*_9_, *Z*_10_, and *Z*_11_ represent the variables of yield, plant height, stem diameter, CP, NAF, ADF, EE, NAE, NUE, PAE, and PUE, respectively. *A*1, *A*2, and *A*3 represent the mass of principal components 1, 2, and 3 in 2019, respectively. *F*_2019_ represents the results of the comprehensive evaluation in 2019.

The mathematical model for the comprehensive evaluation of principal component analysis in 2020 was as follows:


(7) $$\begin{array}{c} f_{4}=0.127\times Z_{1}+0.134\times Z_{2}+0.133\times Z_{3}+0.129\times Z_{4}-0.131\\ \times Z_{5}-0.128\times Z_{6}+0.130\times Z_{7}+0.062\times Z_{8}+0.052\times Z_{9}\\ +0.088\times Z_{10}+0.060\times Z_{11} \end{array}$$

(8) $$\begin{array}{c} f_{2}=0.135\times Z_{1}-0.045\times Z_{2}-0.057\times Z_{3}-0.053\times Z_{4}+0.052\\ \times Z_{5}+0.091\times Z_{6}-0.020\times Z_{7}+0.454\times Z_{8}+0.462\times Z_{9}\\ -0.189\times Z_{10}-0.179\times Z_{11} \end{array}$$

(9) $$\begin{array}{c} f_{3}=0.065\times Z_{1}-0.122\times Z_{2}-0.130\times Z_{3}-0.053\times Z_{4}\\ +0.168\times Z_{5}+0.234\times Z_{6}-0.145\times Z_{7}+0.142\times Z_{8}+0.201\\ \times Z_{9}+0.480\times Z_{10}+0.693\times Z_{11} \end{array}$$

(10) $$F_{2020}=A_{4}\times f_{4}+A_{5}\times f_{5}+A_{6}\times f_{6}$$

In the mathematical model, *f*_4_, *f*_5_, and *f*_6_ represent the score of principal component 1, 2, and 3 in 2020. *A*_4_, *A*_5_, and *A*_6_ represent the mass of principal components 1, 2, and 3 in 2020, respectively. *F*_2020_ represents the results of the comprehensive evaluation in 2020.

**Figure 12:**
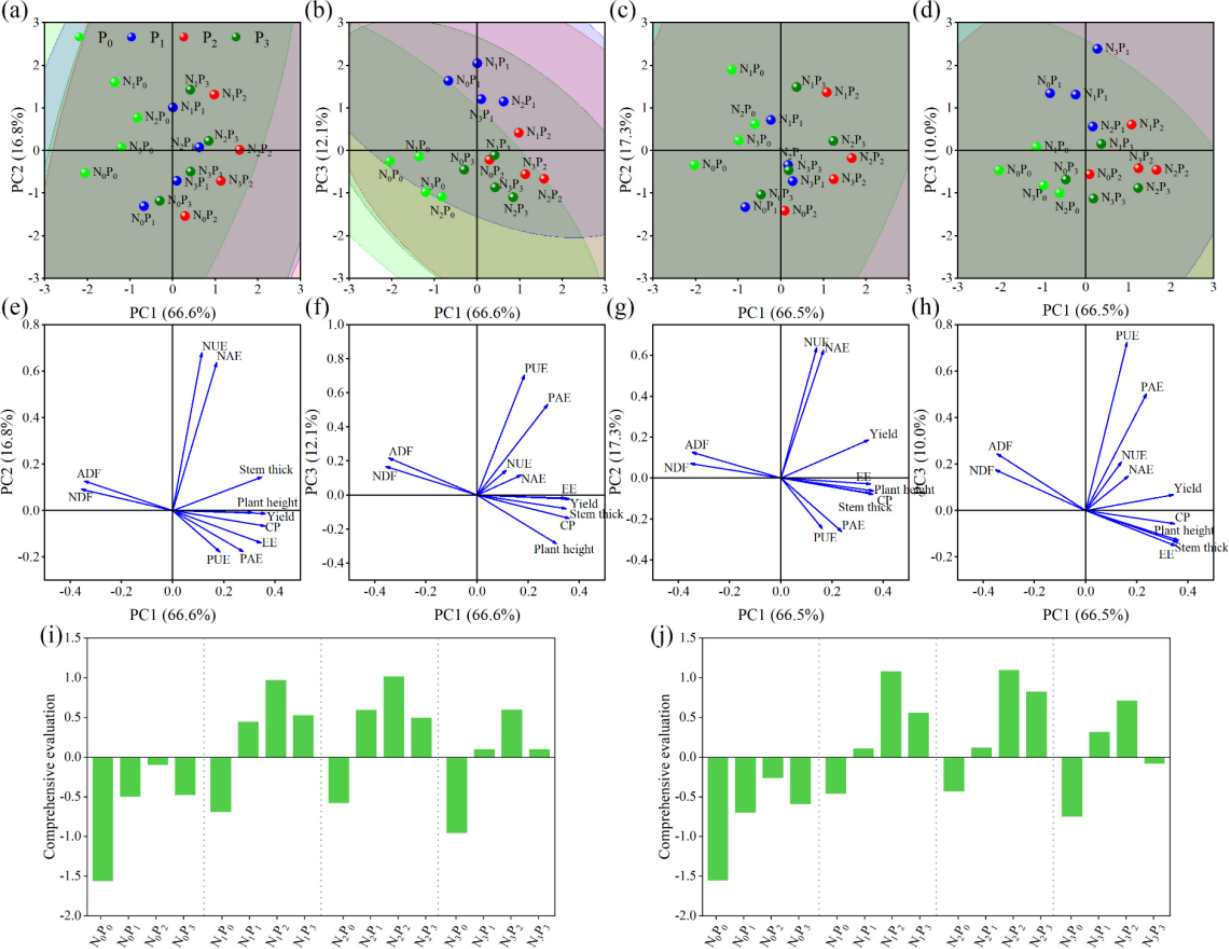
Principal component analysis score map [**(A, B)** in 2019; **(C, D)** in 2020], load map [**(E, F)** in 2019; **(G, H)** in 2020] and comprehensive evaluation [**(I)** in 2019; **(J)** in 2020] of DM yield, growth traits, nutritional quality, and fertilizer use efficiency of alfalfa under different fertilization treatments.

In 2019, the first principal component accounted for 66.6% of the total variance, with yield, CP, NDF, stem thickness, ADF, EE, and plant height contributing the most. The second principal component accounted for 16.8% of the total variance, with NUE and NAE making the largest contributions and plant height making the smallest contribution. The third principal component accounted for 12.1% of the total variance, with PUE and PAE making the largest contributions and EE making the smallest contribution. The cumulative contribution rates of the first, second, and third principal components reached 95.4%, representing the majority of information regarding DM yield, growth traits, and nutritional quality (**Figures 12E, F**; **Supplementary Table S1**). The comprehensive scores of different fertilizer treatments ranked from high to low was N_2_P_2_> N_1_P_2_> N_3_P_2_> N_2_P_1_> N_1_P_3_> N_2_P_3_> N_1_P_1_> N_3_P_3_> N_3_P_1_> N_0_P_2_> N_0_P_3_> N_0_P_1_> N_2_P_0_> N_1_P_0_> N_3_P_0_> N_0_P_0_ (**Figures 12A, B, I**).

In 2020, the first principal component accounted for 66.5% of the total variance, with yield, CP, NDF, stem thickness, EE, ADF, and plant height making the largest contributions. The second principal component accounted for 17.3% of the total variance, with NUE, NAE, PAE, and PUE making the largest contributions and stem thickness making the smallest contribution. The third principal component accounted for 10.0% of the total variance, with PUE, PAE, ADF, and NUE making the largest contributions and EE making the smallest contribution. The cumulative contribution rates of the first, second, and third principal components reached 93.8%, representing the majority of information regarding DM yield, growth traits, and nutritional quality (**Figures 12G, H**; **Supplementary Table S1**). The comprehensive scores of different fertilizer treatments ranked from high to low was N_2_P_2_> N_1_P_2_> N_2_P_3_> N_3_P_2_> N_1_P_3_> N_3_P_1_> N_2_P_1_> N_1_P_1_> N_3_P_3_> N_0_P_2_> N_2_P_0_> N_1_P_0_>N_0_P_3_> N_0_P_1_> N_3_P_0_> N_0_P_0_ (**Figures 12C, D, J**).

### 3.8 Economic benefit

We calculated the total net profit during the experiment (**Table 3**). The investment primarily includes seeds, drip irrigation equipment, Cultivation, irrigation, fertilizers, harvesting, and labor input. The output is the total revenue from the sale of alfalfa hay over two years. The N_2_P_2_ treatment had the highest total net profit of 5978.19 $·ha^-1^ which is a 44.18% increase compared to the non-fertilized treatment. The N_2_P_3_ treatment followed, with a total net profit of 5822.69 $·ha^-1^. The lowest net profit was observed in the N_0_P_0_ treatment, with a total net profit of only 4146.44 $·ha^-1^.

**Table 3:** Total net profit of alfalfa under different fertilization treatments during the experimental period (2019 and 2020).

Treatment	Total income ($·ha^-1^)	Total cost from 2019 to 2020 ($·ha^-1^)							Total net profit ($·ha^-1^)
		Seed	Drip-irrigation facility	Cultivation	Irrigation	Fertilizer	Harvest	Labor	
N_0_P_0_	6973.98	171.86	306.90	143.22	1043.46	16.34	859.32	286.44	4146.44
N_0_P_1_	7468.99	171.86	306.90	143.22	1043.46	86.83	859.32	286.44	4570.96
N_0_P_2_	7809.68	171.86	306.90	143.22	1043.46	157.33	859.32	286.44	4841.14
N_0_P_3_	7722.80	171.86	306.90	143.22	1043.46	227.84	859.32	286.44	4683.76
N_1_P_0_	7743.10	171.86	306.90	143.22	1043.46	44.26	859.32	286.44	4887.63
N_1_P_1_	7982.68	171.86	306.90	143.22	1043.46	114.75	859.32	286.44	5056.73
N_1_P_2_	8792.80	171.86	306.90	143.22	1043.46	185.26	859.32	286.44	5796.34
N_1_P_3_	8544.15	171.86	306.90	143.22	1043.46	255.77	859.32	286.44	5477.18
N_2_P_0_	7844.96	171.86	306.90	143.22	1043.46	72.21	859.32	286.44	4961.55
N_2_P_1_	8115.54	171.86	306.90	143.22	1043.46	142.7	859.32	286.44	5161.63
N_2_P_2_	9002.60	171.86	306.90	143.22	1043.46	213.21	859.32	286.44	5978.19
N_2_P_3_	8917.61	171.86	306.90	143.22	1043.46	283.71	859.32	286.44	5822.69
N_3_P_0_	7696.00	171.86	306.90	143.22	1043.46	100.14	859.32	286.44	4784.66
N_3_P_1_	8526.87	171.86	306.90	143.22	1043.46	170.62	859.32	286.44	5545.05
N_3_P_2_	8791.40	171.86	306.90	143.22	1043.46	241.13	859.32	286.44	5739.06
N_3_P_3_	8126.46	171.86	306.90	143.22	1043.46	311.64	859.32	286.44	5003.62

The price of alfalfa is calculated based on the average purchase price over a two-year period. The drip irrigation facility includes costs associated with the purchase of drip irrigation materials and labor for installation, assessed based on an average lifespan of four years. Cultivation encompasses expenses related to tilling and sowing. Irrigation includes water fees and electricity costs for the operation of the drip irrigation system. Harvest covers expenses for harvesting, drying, bundling, and transportation. Labor comprises costs for weed control, routine management, maintenance of drip irrigation equipment, and other related activities.

## 4 Discussion

### 4.1 Effect of nitrogen and phosphorus rationing on dry matter yield of alfalfa

Aboveground DM yield of forage directly reflects the productivity and overall benefit of forage, which is an important indicator for evaluating forage. In this study, the cumulative DM yield of alfalfa was significantly affected (*P*< 0.01) by the interaction effects of N and P. This result is in line with most previous studies (Mussarat et al., 2021; Xiao et al., 2021). N and P are crucial macronutrients in crop growth, impacting yield formation, quality improvement, and metabolic processes. Among them, N is the main component of proteins, amino acids, and chlorophyll. It is generally believed that soil N content and biological N fixation are two available strategies for alfalfa to obtain N. The addition of exogenous N can effectively increase the effectiveness of soil nutrients, which can transfer the competition pressure among crops from underground to above ground, inducing more resource allocation to the shoots and leaves (Hussain et al., 2018).

Some early literature suggests that as a leguminous plant, alfalfa can form a rhizobial symbiosis through infection of the roots by rhizobia in the soil, and the nitrogenase activity can reduce atmospheric N_2_ to NH_3_, thereby meeting the N requirements for normal alfalfa growth (Zhou et al., 2019). However, addition of a small amount of N fertilizer have been demonstrated to promote alfalfa growth and production. For example, during the initial growth stage and the regeneration period each year, the nitrogenase activity of alfalfa rhizobia symbiosis is weak, and the addition of exogenous N fertilizer can significantly increase the alfalfa DM yield (Kamran et al., 2022). It is thought that N as the growing age of alfalfa increases and the root system develop, alfalfa growth and yield N requirements can be satisfied by N fixed by the rhizobia. This in part, may explain decreased alfalfa persistence and decline in yield commonly observed overtime.

The majority of the agricultural land in Shihezi is characterized as saline-alkali soil, with high salt content, low nutrient levels, and poor structure, which is inadequate for supporting most crop growth (Liu et al., 2021). This is likely the primary reason why N application significantly improves DM yield of alfalfa in this experiment. Under conditions where the soil texture is poor and has not undergone effective improvement, the application of N fertilizer can serve as a measure to increase crop production. In this study, during the first and second years of alfalfa establishment, the root system of alfalfa was not fully developed in the first year, and there was a substantial increase in yield in the second year, resulting in an increased demand for N. Therefore, when compared to the control treatment (no-fertilizer treatment), the N application had a significant impact on the yield of alfalfa.

Phosphorus is also an essential element in plant growth and development, serving as a major component for the synthesis of phospholipids, nucleic acids, ATP, and other substances in plants. It plays an irreplaceable role in physiological processes such as photosynthesis, respiration, carbohydrate metabolism, and N metabolism (Chtouki et al., 2022). In the cultivation of alfalfa, P promotes increased yield by altering the characteristics of yield components. This is consistent with the findings of this study, where P application not only increased the DM yield of alfalfa but also promoted plant height and stem thickness.

Although the P content in alfalfa plants is only 0.2%–0.5%, P plays a significant role in improving alfalfa yield and quality, and the addition of P fertilizer can effectively alleviate P limitations (Yu et al., 2022). Research on the interaction between crop density and P fertilizer application has found that increasing P fertilizer can partially compensate for grain losses when crop density is reduced (Yu et al., 2022). In soil, most available P is immobilized and fixed by the soil, making it difficult to dissolve and transfer. Even in soils with higher P content, only a small amount of P can be absorbed and utilized by plants, and the efficiency of P utilization by plants in the soil is much lower than that of other elements (Wang et al., 2021b). In this study, compared to the no-fertilizer treatment, the interaction between N and P resulted in the highest increase in DM yield of alfalfa by 29.14%. Research has shown that P and N generally have a synergistic effect. After P application, the number of nodules and N fixation activity of alfalfa increase, and the hemoglobin content in the nodules increases, achieving the effect of promoting N with P (Ma and Chen, 2021). The application of N fertilizer can also alter the rhizosphere microenvironment of alfalfa, activating the plant’s uptake of P and increasing P concentration in alfalfa plants. The combined application of N and P plays a critical role in balancing nutrients and had a positive effect on alfalfa growth and increased yield.

### 4.2 Effect of nitrogen and phosphorus rationing on alfalfa nutritional quality

The northern part of Xinjiang is mainly dominated by medium and low cultivated land evaluated as grade 4 to 10 (Ministry of Agriculture and Rural Affairs of the People's Republic of China National Cultivated Land Quality Grades in 2019, 2020), which is characterized by high salt content, prone to desertification, poor soil nutrients, and low productivity. Supplemental application of N and P fertilizers plays a crucial role in maintaining crop nutrient growth and yield. Indicators that reflect the nutritional quality of forage include CP, acidic detergent fiber, NDF, relative feeding value, etc. CP and NDF are the most important nutritional quality indicators for the development of animal husbandry. In this study, P application level had a greater effect than N application level and N-P interaction level in most of the nutritional indicators. Phosphorus plays an important role in promoting high-quality alfalfa growth, while N, although it improves alfalfa nutritional quality, is more capable of nitrogen fixation by the rhizobia with which it is symbiotically associated, and can somewhat compensate for the reduction in externally-added N (Zhou et al., 2019). Studies have shown that fertilization can significantly increase the CP content and reduce the fiber content of red clover. Some studies have also suggested that excessive N fertilizer application promotes the growth of grasses and weeds, thereby affecting the yield, quality, and persistence of alfalfa (Kamran et al., 2022). In this study, CP of alfalfa showed an increasing trend at low and medium fertilizer levels but a decreasing trend at high fertilizer levels.

Excessive N fertilizer application can cause premature flowering in alfalfa, inhibit the root growth, and reduce nutrient absorption capacity. Moreover, high N levels can inhibit the development of rhizobia and reduce their N fixation capacity. Research has shown that both alfalfa yield and quality increase with increasing P application, but there is a saturation point (Liu et al., 2021). Excessive P application can enhance plant respiration, consuming a large amount of carbohydrate and energy, which has a negative impact on plant growth. Additionally, excessive P can lead to an imbalance between aboveground and root growth, with suppressed aboveground growth and highly developed roots.

### 4.3 Effect of nitrogen and phosphorus rationing on alfalfa fertilizer use efficiency

There are various indicators for fertilizer use efficiency, which primarily depend on researchers’ objectives (e.g., economic benefits, nutrient recycling, soil nutrient utilization) and the study level (e.g., agronomy, physiological mechanisms, molecular breeding). Nutrient agronomic use efficiency is used to compare the crop yield with the increase in unit nutrient input, with the goal of guiding appropriate fertilizer application rates to enhance the economic benefits of fertilizer inputs. In this study, as N application rates increased, N agronomic use efficiency showed a gradual decline, while P agronomic use efficiency exhibited an initial increase followed by a decline (except under the P_1_ condition in 2020). With increasing P application rates, P agronomic use efficiency gradually decreased, while N agronomic use efficiency showed an initial increase followed by a decline (except under the N_1_ and N_2_ conditions in 2020). A study on rice found that N agronomic use efficiency was higher under low N conditions when compared to moderate and high N conditions for two consecutive years (Rahman et al., 2014). Although excessive N application did not improve N agronomic use efficiency, it did increased grain agronomic efficiency and N concentration (Rahman et al., 2014).

Studies on P fertilizer also showed that P fertilizer use efficiency is higher when soil P is deficient compared to when it is abundant (Huygens and Saveyn, 2018). Simply increasing fertilizer application rates does not improve fertilizer agronomic use efficiency. Under the same fertilizer application rate, different fertilizer types, application methods, and timing are effective ways to enhance fertilizer use efficiency. Hussain’s research indicated that the N agronomic use efficiency of nitrophos fertilizer was significantly higher than that of urea and nitrate N sources (Hussain et al., 2018). A meta-analysis of global nitrogen application and N agronomic use efficiency showed that the use efficiency of NH_4_^+^ or NH_4_NO_3_ N fertilizer was higher than that of urea. Apart from millet and sorghum, maize had higher N fertilizer use efficiency when compared to other crops (Liang et al., 2022). It is not surprising that N fertilizer use efficiency decreases with increasing N application rates, as excessive N inputs can lead to more severe soil acidification (Sainju and Alasinrin, 2020), nitrate N leaching (Wang et al., 2021b), plant N saturation (Niu et al., 2016), and losses of essential nutrient cations (Ca^2+^, Mg^2+^, Na^+^, Al^3+^, Fe^3+^) (Meng et al., 2019). Studies on P fertilizer have found differences in crop DM yield and P fertilizer use efficiency based on the crop type, P fertilizer type, application timing, and experimental design.

Nutrient uptake efficiency depends on both the nutrient supply capacity and nutrient availability to the root system, as well as the selectivity nutrient uptake and transport capability of plant roots. Similar to fertilizer agronomic use efficiency, the application of fertilizers does not enhance the corresponding fertilizer uptake efficiency, but rather increases the efficiency of non-corresponding fertilizer uptake (Rahman et al., 2014). Increased fertilizer application also reduces the fertilizer uptake efficiency of wheat (Hussain et al., 2018). Nutrient uptake efficiency is closely related to the growth process of alfalfa plants. When the nutrient supply in the soil exceeds the plant’s growth demand, the growth ability of alfalfa plants determines the amount of nutrient uptake. For instance, crop varieties with high N response, often referred to as “high-N type,” tend to accumulate higher N levels in their nutrient and reproductive organs per unit area. However, when crop growth demands for nutrients far exceed the nutrient supply capacity in the soil, the growth potential of plants cannot be fully realized, and nutrient uptake efficiency becomes crucial for nutrient uptake (Huygens and Saveyn, 2018). The greater the adsorption capacity for P, the smaller the fertilizer effect. However, over time, the adsorbed P can be further converted and absorbed by alfalfa. For alfalfa production, it is necessary to consider both the yield-increasing effect of P fertilizer and the residual effect of P fertilizer in the soil in order to achieve rational and economical fertilization (BMPs; Best management practices).

## 5 Conclusions

Two consecutive years of field experiments showed that the P level, the N level, and their interaction had significant (*P<* 0.05) effects on the cumulative DM yield of alfalfa. Compared with no fertilization treatment, the DM yield under fertilization treatment increased by 7.43% - 29.4%. Additionally, fertilization increased alfalfa plant height, stem thickness, CP, and ether extract, while decreasing NDF and ADF. Although fertilization reduced the corresponding fertilizer use efficiency, evidence from our research suggests that fertilization can enhance the non-corresponding fertilizer use efficiency. when urea was applied at 286.3 kg ·ha^-1^ and monoammonium phosphate at 192 kg·ha^-1^, the comprehensive evaluation of growth indicators and fertilizer use efficiency were optimal. Simultaneously, the highest total net profit reached 5978.19 $·ha^-1^, representing a 44.18% increase compared to the non-fertilized treatment. This study provides a theoretical basis for managing subsurface drip fertigated alfalfa in northern Xinjiang.

## Data Availability

The original contributions presented in the study are included in the article/**Supplementary Material**. Further inquiries can be directed to the corresponding author/s.

## References

[B1] Anas M. Liao F. Verma K. K. Sarwar M. A. Mahmood A. Chen Z.-L.. (2020). Fate of nitrogen in agriculture and environment: agronomic, eco-physiological and molecular approaches to improve nitrogen use efficiency. Biol. Res. 53, 47. doi: 10.1186/s40659-020-00312-4 33066819 PMC7565752

[B2] AOAC (2005). “Official methods of analysis of the association of official analytical international,” in Method 989.05, 18th ed. Ed. Horwitz W. (AOAC International, Arlington, USA).

[B3] Bastos L. M. Carciochi W. Lollato R. P. Jaenisch B. R. Rezende C. R. Schwalbert R.. (2020). Winter wheat yield response to plant density as a function of yield environment and tillering potential: A review and field studies. Front. Plant Sci. 11. doi: 10.3389/fpls.2020.00054 PMC7066254 32194579

[B4] Bhandari K. B. West C. P. Acosta-Martinez V. (2020). Assessing the role of interseeding alfalfa into grass on improving pasture soil health in semi-arid Texas High Plains. Appl. Soil Ecol. 147, 103399. doi: 10.1016/j.apsoil.2019.103399

[B5] Chtouki M. Laaziz F. Naciri R. Garré S. Nguyen F. Oukarroum A. (2022). Interactive effect of soil moisture content and phosphorus fertilizer form on chickpea growth, photosynthesis, and nutrient uptake. Sci. Rep. 12, 6671. doi: 10.1038/s41598-022-10703-0 35461340 PMC9035189

[B6] Elgharably A. Benes S. (2021). Alfalfa biomass yield and nitrogen fixation in response to applied mineral nitrogen under saline soil conditions. J. Soil Sci. Plant Nutr. 21, 744–755. doi: 10.1007/s42729-020-00397-6

[B7] Fan J. Hao M. Malhi S. S. Wang Q. Huang M. (2011). Influence of 24 annual applications of fertilisers and/or manure to alfalfa on forage yield and some soil properties under dryland conditions in northern China. Crop Pasture Sci. 62, 437. doi: 10.1071/CP10370

[B8] Hussain M. Cheema S. A. Abbas R. Q. Ashraf M. F. Shahzad M. Farooq M.. (2018). Choice of nitrogen fertilizer affects grain yield and agronomic nitrogen use efficiency of wheat cultivars. J. Plant Nutr. 41, 2330–2343. doi: 10.1080/01904167.2018.1509998

[B9] Huygens D. Saveyn H. G. M. (2018). Agronomic efficiency of selected phosphorus fertilisers derived from secondary raw materials for European agriculture. A meta-analysis. Agron. Sustain. Dev. 38, 52. doi: 10.1007/s13593-018-0527-1

[B10] Kamran M. Yan Z. Jia Q. Chang S. Ahmad I. Ghani M. U.. (2022). Irrigation and nitrogen fertilization influence on alfalfa yield, nutritive value, and resource use efficiency in an arid environment. Field Crops Res. 284, 108587. doi: 10.1016/j.fcr.2022.108587

[B11] Liang G. Sun P. Waring B. G. (2022). Nitrogen agronomic efficiency under nitrogen fertilization does not change over time in the long term: Evidence from 477 global studies. Soil Tillage Res. 223, 105468. doi: 10.1016/j.still.2022.105468

[B12] Lin S. Pi Y. Long D. Duan J. Zhu X. Wang X.. (2022). Impact of organic and chemical nitrogen fertilizers on the crop yield and fertilizer use efficiency of soybean–maize intercropping systems. Agriculture 12, 1428. doi: 10.3390/agriculture12091428

[B13] Liu Y. Wu Q. Ge G. Han G. Jia Y. (2018). Influence of drought stress on alfalfa yields and nutritional composition. BMC Plant Biol. 18, 13. doi: 10.1186/s12870-017-1226-9 29334916 PMC5769550

[B14] Liu X. Zhao J. Liu J. Lu W. Ma C. Gu X.. (2021). Water–phosphorus coupling enhances fine root turnover and dry matter yield of alfalfa under drip irrigation. Agron. J. 113, 4161–4175. doi: 10.1002/agj2.20782

[B15] Ma Y. Chen R. (2021). Nitrogen and phosphorus signaling and transport during legume–rhizobium symbiosis. Front. Plant Sci. 12. doi: 10.3389/fpls.2021.683601 PMC8258413 34239527

[B16] Malhotra H. Vandana Sharma S. Pandey R. (2018). Phosphorus nutrition: plant growth in response to deficiency and excess, plant nutrients and abiotic stress tolerance (Singapore: Springer Singapore). doi: 10.1007/978-981-10-9044-8_7

[B17] Meng C. Tian D. Zeng H. Li Z. Yi C. Niu S. (2019). Global soil acidification impacts on belowground processes. Environ. Res. Lett. 14, 074003. doi: 10.1088/1748-9326/ab239c

[B18] Ministry of Agriculture and Rural Affairs of the People's Republic of China National Cultivated Land Quality Grades in 2019 (2020). Available online at: http://www.ntjss.moa.gov.cn/zcfb/202006/P020200622573390595236.pdf (Accessed January 14, 2023).

[B19] Mussarat M. Shair M. Muhammad D. Mian I. A. Khan S. Adnan M.. (2021). Accentuating the role of nitrogen to phosphorus ratio on the growth and yield of wheat crop. Sustainability 13, 2253. doi: 10.3390/su13042253

[B20] Niu S. Classen A. T. Dukes J. S. Kardol P. Liu L. Luo Y.. (2016). Global patterns and substrate-based mechanisms of the terrestrial nitrogen cycle. Ecol. Lett. 19, 697–709. doi: 10.1111/ele.12591 26932540

[B21] Putra R. Powell J. R. Hartley S. E. Johnson S. N. (2020). Is it time to include legumes in plant silicon research? Funct. Ecol. 34, 1142–1157. doi: 10.1111/1365-2435.13565

[B22] Rahman M. M. Islam A. M. Azirun S. M. Boyce A. N. (2014). Tropical legume crop rotation and nitrogen fertilizer effects on agronomic and nitrogen efficiency of rice. Sci. World J. 2014, e490841. doi: 10.1155/2014/490841 PMC4055622 24971378

[B23] Sainju U. M. Alasinrin S. Y. (2020). Changes in soil chemical properties and crop yields with long-term cropping system and nitrogen fertilization. Agrosystems Geosciences Environ. 3, e20019. doi: 10.1002/agg2.20019

[B24] Song X. Fang C. Yuan Z. Q. Li F. M. Sardans J. Penuelas J. (2022). Long-term alfalfa (*Medicago sativa* L.) establishment could alleviate phosphorus limitation induced by nitrogen deposition in the carbonate soil. J. Environ. Manage. 324, 116346. doi: 10.1016/j.jenvman.2022.116346 36166863

[B25] Sun Y. Wang X. Ma C. Zhang Q. (2022). Effects of nitrogen and phosphorus addition on agronomic characters, photosynthetic performance and anatomical structure of alfalfa in northern Xinjiang, China. Agronomy 12, 1613. doi: 10.3390/agronomy12071613

[B26] Van Soest P. J. Robertson J. B. Lewis B. A. (1991). Methods for dietary fiber, neutral detergent fiber, and nonstarch polysaccharides in relation to animal nutrition. J. Dairy Sci. 74, 3583–3597. doi: 10.3168/jds.S0022-0302(91)78551-2 1660498

[B27] Vurro F. Manfredi R. Bettelli M. Bocci G. Cologni A. L. Cornali S.. (2023). *In vivo* sensing to monitor tomato plants in field conditions and optimize crop water management. Precis. Agric. 24, 2479–2499. doi: 10.1007/s11119-023-10049-1

[B28] Wan W. Liu Q. Zhang C. Li K. Sun Z. Li Y.. (2023). Alfalfa growth and nitrogen fixation constraints in salt-affected soils are in part offset by increased nitrogen supply. Front. Plant Sci. 14. doi: 10.3389/fpls.2023.1126017 PMC9989181 36895871

[B29] Wang Y. Li M. Yan H. (2021b). Ammonia volatilization from urea in alfalfa field with different nitrogen application rates, methods and timing. Agriculture Ecosyst. Environ. 312, 107344. doi: 10.1016/j.agee.2021.107344

[B30] Wang L. Zheng J. You J. Li J. Chen Q. Leng S.. (2021a). Effects of phosphorus supply on the leaf photosynthesis, and biomass and phosphorus accumulation and partitioning of canola (*Brassica napus* L.) in saline environment. Agronomy 11, 1918. doi: 10.3390/agronomy11101918

[B31] Xia D. An X. López I. F. Ma C. Zhang Q. (2023). Enhancing alfalfa photosynthetic performance through arbuscular mycorrhizal fungi inoculation across varied phosphorus application levels. Front. Plant Sci. 14. doi: 10.3389/fpls.2023.1256084 PMC10623315 37929180

[B32] Xiao J. Zhu Y. Bai W. Liu Z. Tang L. Zheng Y. (2021). Yield performance and optimal nitrogen and phosphorus application rates in wheat and faba bean intercropping. J. Integr. Agric. 20, 3012–3025. doi: 10.1016/S2095-3119(20)63489-X

[B33] Yu Q. Ni X. Cheng X. Ma S. Tian D. Zhu B.. (2022). Foliar phosphorus allocation and photosynthesis reveal plants’ adaptative strategies to phosphorus limitation in tropical forests at different successional stages. Sci. Total Environ. 846, 157456. doi: 10.1016/j.scitotenv.2022.157456 35863563

[B34] Zhou J. Wilson G. W. T. Cobb A. B. Yang G. Zhang Y. (2019). Phosphorus and mowing improve native alfalfa establishment, facilitating restoration of grassland productivity and diversity. Land Degrad Dev. 30, 647–657. doi: 10.1002/ldr.3251

[B35] Zielewicz W. Grzebisz W. Przygocka-Cyna K. Goliński P. (2023). Productivity of nitrogen accumulated in alfalfa–grass sward cultivated on soil depleted in basic nutrients: a case study. Agronomy 13, 1765. doi: 10.3390/agronomy13071765

