# Supplementary material for: Nitrogen and phosphorus fertilizer use efficiency improves alfalfa (Medicago sativa L.) production and performance in alkaline desert soil
Source: Front Plant Sci. 2025 Feb 18;16:1526648. doi: 10.3389/fpls.2025.1526648 (PMC11876427; doi:10.3389/fpls.2025.1526648)
Supplement: Supplementary file 1 [file Table1.docx]

**Supplementary Information (SI)**

**for**

**Nitrogen and Phosphorus Fertilizer Use Efficiency Improves Alfalfa (*Medicago sativa* L.) Production and Performance in Northern Xinjiang, China**

Yanliang Sun^1^, Jing Sun^1^, Xuzhe Wang^1^, Andrew D. Cartmill^2^, Ignacio F. López^2^, Chunhui Ma^1^* and Qianbing Zhang^1^*

^1^ College of Animal Science and Technology, Shihezi University, Shihezi, Xinjiang, China.

^2^ School of Agriculture and Environment, Massey University, Palmerston North 4442, New Zealand

Table S1 Eigenvalues and variance contribution rates of principal component analysis

| Principal component | 2019 Initial eigenvalue | | |  | 2020 Initial eigenvalue | | |
| --- | --- | --- | --- | --- | --- | --- | --- |
|  | Eigenvalue | Variance contribution rate | Cumulative contribution rate |  | Eigenvalue | Variance contribution rate | Cumulative contribution rate |
| 1 | 7.32369 | 66.57898 | 66.57898 |  | 7.31476 | 66.49784 | 66.49784 |
| 2 | 1.84635 | 16.78502 | 83.36401 |  | 1.9062 | 17.32907 | 83.82691 |
| 3 | 1.32577 | 12.0525 | 95.41651 |  | 1.09488 | 9.95348 | 93.78039 |
| 4 | 0.35473 | 3.22479 | 98.6413 |  | 0.37536 | 3.41233 | 97.19271 |
| 5 | 0.05322 | 0.48385 | 99.12515 |  | 0.12239 | 1.1126 | 98.30531 |
| 6 | 0.04416 | 0.40146 | 99.52661 |  | 0.08328 | 0.75712 | 99.06243 |
| 7 | 0.03721 | 0.33825 | 99.86486 |  | 0.05723 | 0.52023 | 99.58267 |
| 8 | 0.00938 | 0.08528 | 99.95014 |  | 0.02847 | 0.25878 | 99.84145 |
| 9 | 0.00318 | 0.02891 | 99.97905 |  | 0.01039 | 0.09449 | 99.93593 |
| 10 | 0.00136 | 0.01233 | 99.99139 |  | 0.00468 | 0.04258 | 99.97851 |
| 11 | 0.00095 | 0.00861 | 100 |  | 0.00236 | 0.02149 | 100 |
